# ‘Not my dream’: Mother’s challenge of raising intellectual disability child in Vhembe district

**DOI:** 10.4102/hsag.v27i0.1995

**Published:** 2022-11-15

**Authors:** Ndidzulafhi S. Raliphaswa, Mary Maluleke, Mutshinyalo L. Netshikweta

**Affiliations:** 1 Department of Advanced Nursing Science, Faculty of Health Sciences, University of Venda, Thohoyandou, South Africa

**Keywords:** challenge, intellectual disability, mother, raise, child

## Abstract

**Background:**

The birth of a child with an intellectual disability in a family brings confusion, depression and frustrations, especially to the mother. Activities required by these children place a heavy burden on mothers in terms of support and care, as most mothers are the ones who take full care in the well-being of a child, regardless of whether a child has an intellectual disability or not. Challenges include feeding, bathing, dressing, finance and assisting with elimination, especially when the child is a teenager, where one expects the child to be able to do everything for himself or herself.

**Aim:**

To explore and describe the challenges experienced by mothers of children with intellectual disabilities.

**Setting:**

The study was conducted in the selected hospitals of the Vhembe district in Limpopo province.

**Methods:**

A qualitative, exploratory, descriptive and contextual research design was adopted. A nonprobability purposive sample comprised 13 mothers of children with intellectual disabilities such as down syndrome and cerebral palsy. Data were collected through unstructured interviews until saturation was reached. Data were analysed by the open-coding method.

**Results:**

Study findings revealed that mothers of children with intellectual disabilities experienced feelings of fear, embarrassment and financial burdens because of children’s special needs and initial awareness of the diagnosis.

**Conclusion:**

It is imperative that mothers of children with intellectual disabilities be supported by family, relatives, health care professionals and the community.

**Contribution:**

The study’s contribution was to strengthen psychological intervention and support to mothers and awareness to accept children with intellectual disability.

## Introduction

Intellectual disability is a general term used to describe children who develop and learn more slowly than children without the disability. Hence, they struggle to survive in their personal and social lives without support. Moreover, it should be noted that daily care for children with intellectual disabilities is different from that of children without disabilities. This is because of the special care that needs to be provided. Children with intellectual disabilities are dependent on someone else for daily living activities (Burns et al. 2019). This situation tends to cause families of children with intellectual disabilities to experience difficulties regarding the needed care (Barr, Govender & Rencken 2016). In Africa, people generally feel good or well when married, as marriage is thought to bring happiness. Furthermore, women became happy when they are able to conceive and give birth to a healthy child. Culturally, the expectation is that being a respected woman, one must give birth to a child without any form of disability (Long & Bullare 2020). This is believed to be able to save an unhappy marriage and solve emotional problems. These expectations bring disappointments to parents, including the father, if the child is born with an intellectual disability (Marsh, Warren & Savage 2018).

Furthermore, Burack et al. (2021) highlighted the developmental approach to intellectual disability that leads to an essential deconstruction in applied theories and methodologies in the field. However, this leads to a more precise but comprehensive understanding. Some effects related to the likelihood that a child will display the cognitive and behavioural phenotypes considered to be typical of children with intellectual disability. These include specific patterns of strengths and weaknesses in information processing, social interaction, expressive language, receptive skills, motor skills and motivation (Albert et al. 2021).

Because of the child’s behaviour, mothers of these children experienced problems raising them, leading to maternal stress as they are always busy with either the child or household routine (Dieleman et al. 2018). The severity of the disorder and social support play significant roles in family quality of life.

Page et al. (2020) also revealed the challenges faced by caregivers caring for children who require medical care. Mothers, as primary caregivers, know their children better and are to give the health care professionals the necessary information based on their observation to help their children with medical issues. However, they tend to have challenges as they are expected to know everything regarding the care of their children with intellectual disabilities, especially when a child is admitted in a hospital. Despite challenges they face, some mothers displayed a positive attitude of being empowered and perceive this as a good opportunity to improve coping strategies. They tend to be closer to each other as a family when caring for this child (Van Esch et al. 2018). On the other hand, the positive feeling revolves around the psychological need for autonomy, relatedness and competence on the side of the mother. However, these mothers tend to be frustrated if this satisfaction is not met, especially when mostly deserted by husbands and lovers. Some mothers are excluded in the community, and their exclusion from social activities by the community members make them withdraw from the world (Ryan & Deci 2017). Given all that is discussed above, the researchers saw a need to further explore the challenges of mothers parenting children with intellectual disabilities.

### Problem statement

Mothers play a very crucial role in the upbringing of children, with or without disabilities. However, those with intellectual disabilities add more pains and frustrations, as there are a number of activities to be done for those children as compared to children without intellectual disabilities; hence, the activities tend to be stressful (Dabrowska & Pisula 2010). The researchers observed that most mothers of children with intellectual disabilities were displaying some difficulties with the care of their children. During a child’s hospitalisation, mothers were seen to be emotional, reserved and isolated. When engaging with some of the mothers, they indicated the lack of support, discrimination and isolation from family members and community. In view of this, the researchers saw a need to explore more on the challenges faced by the mothers of these children and to provide recommendations that might assist to support them based on the research findings.

### Study purpose

The purpose of this study was to understand the challenges experienced by mothers of parenting a child with intellectual disabilities in the selected hospitals of Vhembe district.

## Research methods and design

A qualitative, descriptive, exploratory and contextual research design was used (De Vos et al. 2012). This enables the researchers to explore and describe the challenges faced by mothers when parenting children, which was also seen to be appropriate as little is known about the topic under study. The participants were able to express with intellectual disabilities in Vhembe district of Limpopo province. The research design was chosen as it enables the researchers to get rich information as experienced by the mothers. The design explored their feelings and experiences as lived-in real-life situations.

### Setting

The study was conducted in the selected hospitals of the Vhembe district. Vhembe district is in a deep rural area of Limpopo province in South Africa, which consists of four local municipalities, that is, Thulamela, Collins Chabane, Makhado and Musina municipalities. There are two hospitals in Thulamela municipality, Tshilidzini and Donald Fraser Hospitals; in Makhado municipality, there are three hospitals, Siloam, Elim and Louis Tritchardt Hospitals; Musina has one hospital, Messina Hospital, and Collins Chabane has Malamulele Hospital. The population consisted of 13 mothers whose children with intellectual disabilities were admitted in the selected paediatric medical wards.

### Sampling method

Nonprobability purposive sampling method was used to sample 13 mothers who are parenting a child with an intellectual disability and were willing to participate in the study and share their challenges (Polit & Beck 2017). Purposive sampling was chosen as this study was focusing on only mothers of children with intellectual disabilities. The inclusion criteria were mothers aged 26–30 years whose children were between 6 and 12 years of age and who were willing to participate, share their knowledge, sign an informed consent form and be included in the study. Mothers who were not mentally stable, not willing to participate and not within the specified age limit were excluded.

### Data collection

Data were collected by the main researcher using unstructured in-depth individual interviews from the participants who were willing to participate and signed the consent form. The data collection method was chosen as it encourages the participants to talk freely about the topic that is being explored. The question that guided the interview was, ‘May you kindly share with me challenges of parenting your child with an intellectual disability?’ Probing was done to elicit more information from the participants. This was done after the pilot study in order to identify possible flaws, such as the researcher’s probing skills, which were improved after the pilot study. Pilot study was done from the population with the same inclusion criteria as the main study, and only three participants were part of the pilot study. Participants who were part of the pilot study were not included in the main study, but information obtained was included as it was relevant and made the data richer.

A voice recorder was used to capture and record the findings after permission was granted by the participants. This was done to capture everything said by the participants to avoid missing out on some important information. Collected data from recordings were transcribed verbatim and translated to English as the interviews were conducted in the participant’s home language, which was Tshivenda and Xitsonga (De Vos et al. 2013). This was done to capture everything said by the participants to avoid missing out on some important information. Transcripts were sent back to the participants to ensure that what was transcribed is the true reflection of what the participants have said. This was done so that participants may have an opportunity to comment or make corrections if there was an error in transcription. Field notes were taken during the interviews.

Data were collected for 3 months, and each interview lasted for 30 min to 45 min. Data saturation was reached at the eighth participant. However, the researcher went on with the interviews by adding five participants to ensure that indeed nothing new was coming out. The interviews focused on mothers’ challenges of parenting children with intellectual disabilities.

### Data analysis

The researchers used open coding according to Tesch’s inductive, descriptive coding technique (in Creswell 2009), quoted in Botman et al. (2010), for data analysis. The narrative data from the in-depth interviews were analysed qualitatively. The method included the following steps: the researcher read carefully through all the transcripts to get a sense of whole. After the completion of all transcripts, a list of similar topics was compiled. Researchers classified the qualitative information by looking for categories, themes or dimensions of information. Then data were grouped according to themes and subthemes. Field notes were also coded and categorised. An independent coder was used, and a consensus was reached on the themes and subthemes constructed.

### Trustworthiness

Trustworthiness is a method of establishing rigor in qualitative research without sacrificing relevance (Lincoln & Guba 1985). Rigour assists the researcher in preventing errors. The credibility of the study findings was ensured by using an in-depth individual interview; there was prolonged engagement of the participants during the interviews, which enabled the researchers to collect all the information until data saturation was achieved. Member checking was done by returning to the participants with the transcribed information so that the participants could confirm that indeed the captured data are exactly what they said.

To ensure the transferability of the study findings, documentation was done in simple language regarding how the theme was identified and how subcategories were induced. Transferability was also ensured by clearly providing a detailed description of all research processes, and if other researchers should repeat the same study in another setting, it would come up with the same findings or conclusions.

The researchers used the strategy of confirmability to ensure neutrality. In other words, the researcher avoided bias by remaining neutral. This was ensured through listening to the tapes to verify interpretations, conclusions and recommendations. An audit trail was developed by documenting field notes and voice recordings that allowed an independent coder to reach conclusions about the data. Field notes were checked and compared with nonverbal cues.

Dependability of the study findings was ensured by recording all detailed study processes and documentation for others to replicate. This was ensured through conducting an enquiry audit and scrutinising the data. The researchers used a coding and recoding procedure. The researcher waited for 2 weeks after coding the data and returned to recode the same data and compare the results. An independent coder assisted in data analysis

### Ethical considerations

Ethical clearance was obtained from the University of Venda Research Ethics Committee (reference number: SHS/16/PDC/07/2804), Department of Health Limpopo Province Research Committee, the chief executive officers of the hospitals, district managers of the district hospitals and nurse managers of the hospitals. For ethical purposes, participants were assured that participation in the study was voluntary and that no remuneration or reward would be awarded. Researchers are bound by ethical morals and rules when conducting the research (De Vos et al. 2012). In this study, ethical considerations were adhered to, and the core principles like autonomy and confidentiality were discussed with participants prior to data collection. The ethical considerations were in accordance with statutory ethical standards and the following principles were adhered to: participants were given a consent form to sign, and signing was voluntary; each participant had the right to withdraw at any time, and no penalty was to be given. Fortunately, in this study, all participants participated to the end.

In this study, researchers tried to minimise harm and discomfort by providing participants with the information and the rights to withdraw from the study at any time if not feeling comfortable. Further assistance of counselling was to be provided to those who might become emotional, and arrangements with the psychologist were made for referral purposes.

In this study, researchers kept the records safe and a copy was available to the researchers only.

Anonymity is the protection of participants’ confidentiality such that even the researcher cannot link individuals with the data they provide (Polit & Beck 2017). For anonymity, participants’ real names were not used; instead, each participant was given a pseudonym.

The researchers never withheld information or offered incorrect information to the participants. This was done by explaining to the participants how the interview would be conducted and that there would be no incentives given to the participants.

## Results

The study had 13 participants, and their children’s ages were below 12 years. Data were collected in Vhembe district, Limpopo province. Data were saturated at the eighth participant. Demographic profiles of the participants were presented. Two participants were employed but not receiving enough to be able to support themselves and their children financially. Four participants were not employed and depended on their child’s grant, selling food and clothes to augment the child’s grant they were receiving. Two participants were financially supported by their husbands. Three were not getting any support, including a child’s grant. Two mothers had obtained primary education, six had secondary education and four had tertiary education. Participants (P) were interviewed in Tshivenda and Tsonga as these were their mother tongues, which were translated verbatim into English by the researcher. From the total number of participants, four spoke Tsonga and nine Tshivenda, as illustrated by Table 1.

**TABLE 1:** Demographic profiles of the participants.

Mother’s age (years)	Child’s age (years)	Number of children	Language	Educational level	Support system	Participant number
27	10	2	Tshivenda	Secondary	No support	1
26	6	1	Tshivenda	Tertiary	Child’s grant	2
30	8	2	Tshivenda	Secondary	Self	3
25	7	2	Tshivenda	Secondary	Husband	4
26	11	1	Tshivenda	Secondary	Husband	5
28	6	1	Tshivenda	Primary	No support	6
29	9	1	Tsonga	Secondary	Child’s grant	7
26	6	2	Tsonga	Tertiary	Child’s grant	8
30	6	3	Tshivenda	Tertiary	Self	9
27	8	2	Tsonga	Secondary	Child’s grant	10
27	10	1	Tshivenda	Tertiary	Employed	11
26	7	2	Tshivenda	Tertiary	Employed	12
30	11	1	Tsonga	Primary	No support	13

Research results outlined three themes and six subthemes that emerged during data analysis. Tesch’s open coding method (Creswell 2014) was used to analyse data obtained from 13 mothers of children with intellectual disabilities in Vhembe district, Limpopo province. The emerged themes from the interviews are shown in Table 2.

**TABLE 2:** Themes and subthemes reflecting mothers’ challenges of parenting children with intellectual disabilities in Vhembe district, Limpopo province.

Main themes	Subthemes
1. Experiences related to parenting a child with intellectual disabilities	1.1 Existence of feelings of fear, anger, blaming oneself 1.2 Suspicion and embarrassment, which cause suffering throughout the process of care 1.3 Dehumanising feeling
2. Financial burden experienced because of children special needs	2.1 Distance travelled 2.2 Appointments and follow-up visits with the health care professionals
3. Delayed initial awareness of the diagnosis by the mother	3.1 Denial versus acceptance

Three themes and their related six subthemes will be explained briefly and discussed in detail in the discussion section.

### Theme 1: Experiences related to parenting a child with an intellectual disability

Every child needs support from their parents, relatives and community as a whole despite the condition that the child might have. If parents are not getting any support, they get frustrated and further frustrate a child.

#### Subtheme 1.1: Existence of feelings of fear, anger and blaming oneself

Feelings of fear, anger and blaming oneself have been displayed by mothers of children with intellectual disabilities. This was because of the lack of support from partners, community and relatives. Having a child with intellectual disabilities also caused frustration to the mother. This was because of the risk attached to the weakness and helplessness of a child. This was confirmed by a participant who said:

‘Eish [*looking down*] … She also likes moving around the streets. Once she can go out of the yard and you did not notice that, it is very difficult to find her. It is very bad as these days, the world is not like some years back. There is a lot of bad things happening in our area. I am afraid she might be raped or even killed.’ (P5, 26 years old, 1 child)

Another participant said:

‘… What must I do! Yes, I am always with her. All the events that occur in the community if I am to go, I go with her, though people will always look at us. I may rather leave my youngest child at home and go with this one, because I know she needs more attention from me, and nobody can handle her better than I do because I know her.’ (P2, 26 years old, 1 child)

A participant expressed a feeling of blaming herself by saying:

‘Maybe it is a punishment from God, and that is what God gave me; I cannot kill her. That is one thing that makes me to be always stressful. I was taken to the psychologist, but as this problem continues, it affects me more.’ (P6, 28 years old, 1 child)

Marian, Magesa and Fillipine (2020) shared the same sentiment with the current study by highlighting that mothers were shocked, depressed and frustrated when they heard about their children’s condition.

#### Subtheme 1.2: Suspicion and embarrassment that cause suffering throughout the process of care

Most mothers suffer from feelings of suspicion and embarrassment. The embarrassment caused by people around them instil suspicion and fear to have another child. The study revealed that some mothers were traumatised by having a child with intellectual disability in such a way that they do not even want to have another child. Participant 3 (30 years old, 2 children) stated, ‘what if I get the same child like this one … I am so scared’.

Another mother added:

‘… She is 6 years and I cannot even think of having another child, as I cannot afford to have two disabled infants, as she is also like an infant who needs all my attention. What if I get another child with this condition? [*crying*].’ (P1, 27 years old, 2 children)

She went on and said:

‘… I was telling my husband as he was forcing me to have another child, but I cannot and he is angry with me about that. What must I do then? [*crying*].’ (P1, 27 years old, 2 children)

Mothers felt embarrassed when walking around with their children, adding more burden to them. This was expressed by mothers whose children with intellectual disabilities were the first or second child.

One mother had this to say:

‘She was trying to laugh at us and made us feel very bad. Those children attending special schools are regarded as “mad” children and are not accepted by some community members. It is so embarrassing. It is just that you do not know how I feel because everywhere I go, we tend to be the centre of attraction …. yeah.’ (P7, 29 years old, 1 child)

#### Subtheme 1.3: Dehumanised feeling

Mothers in this study felt that their children were dehumanised as they were not treated as humans by some of the community members. One mother verbalises this angrily:

‘My neighbour has a 4-year-old child and my child like to play with her, but her mother would not allow them to play together. She only allows them to play when she sees me but when she does not see me, they do not play together. My other neighbour also witnessed it and told me not to allow my child to go and play with that child again, as my child is not allowed to touch anything there. She will take a mat that she puts on the floor for her dog and let my child to sit there as if she is a dog too. It is so embarrassing. Ooh, I never thought … [*crying bitterly*].’ (P10, 27 years old, 2 children)

Cage, Di Monaco and Newell (2019) highlighted that people with intellectual disabilities were undermined and not taken as useful people. This was related to abilities and skills, and the medical model discourse often centred around them. Capozza et al. (2016) concur by discussing the human uniqueness of individuals with intellectual disabilities. Their arguments were related to the common misconceptions and underestimations regarding abilities of people with intellectual disabilities. In this study, dehumanisation of people with intellectual disabilities may also relate to such underestimation.

### Theme 2: Financial burden experienced because of children special needs

Many mothers who participated in this study had financial challenges, as their children demanded a good deal from their pockets in relation to their condition. This theme has two subthemes, namely distance travelled and appointments and follow-up visits with the health care providers.

#### Subtheme 2.1: Distance travelled

Mothers in this study revealed the need to be supported financially, as they have little or no support system. The distance that some mothers travel when going to the health facilities is more than a walking distance (> 5 km). Some mothers depend on the South African child’s grant for a living, which is very little.

A mother, whose child is 9 years, said:

‘The distance is too long. I wish I had a car. He does not want to walk, especially long distances. I am forced to put him on my back so that I can catch a bus in the morning when going for a check-up. Even if he is willing to walk, can he really manage to walk such a distance? [*frowning*].’ (P8, 26 years old, 2 children)

#### Subtheme 2.2: Appointments and follow-up visits with the health care professionals

Most of the mothers who participated in this study were not working, resulting in financial constraints. Some depend on their children’s grants, whereas others do not receive a grant and there was no other means of financial support:

‘I just try to use the little money I get from her grant for transport, food and clothes, as I am not working. Sometimes the doctor will need to see her twice a month and I will try to come, even if the money is not enough. The occupational therapist will have his [*or*] her own appointment on the date not similar to that one of the doctor, and I must comply. Even if I want to use this money for something else I cannot, because I value the health of my child first.’ (P4, 25 years old, 2 children)

A participant said:

‘No, I am not working. This up and down is stressing and tiring. Even if I can get a job, my mind will always be in my child, and I only work for few hours and go home. I cannot work for a full day, and I end up leaving a job. I need to take him to the hospital for check-up every month, and my boss, yoh, she does not understand giving me a leave. On the other hand, I need money to take care of him. It is not easy to leave him with somebody else. In fact, who is that person who can take care of him better than I do? Is very difficult … eish.’ (P6, 28 years old, 1 child)

A participant had this to narrate:

‘… The treatment [*or*] support is not right, because sometimes I come here for the check-up of my child but returned home without the child being seen by the doctor because the file will be missing. Sometimes I would come at 06:00 in the morning and go home at 19:00 without the child’s treatment. It is so unfortunate because without the treatment, my child cannot cope. When I try to say it out, I will be told to keep quiet and sit down … Oh, I never thought! The following morning I must come again, using my money which I don’t have, because I will be worried about my child’s medication. Like today, I came at 06:30 and I only got my file now at 12:00, which is not fair. It is like we mothers with intellectual disability children are not treated well, we are taken for granted … [*looks very emotional*].’ (P13, 30 years old, 1 child)

The study revealed that mothers were not happy about how the health care services were rendered to them, as they were not assisted as expected. The primary role for the health care professionals is to provide quality patient care and with good intentions.

### Theme 3: Delayed initial awareness of diagnosis to the mother

Some mothers complained that they were not told about the diagnosis of a child immediately. They indicated that initial awareness was very important to them. This could provide a better understanding to mothers and help them to know what to expect from their children, including their future plans. Every parent expects a healthy normal child when she is pregnant. As a participant said:

‘I knew about her diagnosis when she was 1.5 years [*18 months*]. I was told by the doctor who was treating my child, after I have asked exactly what the problem with the child is, because time and again I was told to bring the child for check-up.’ (P2, 26 years old, 1 child)

Communication by the health care professionals was also a concern to mothers. This was confirmed by a participant who said:

‘It was discovered by his grandmother because the child couldn’t talk and looks very weak, and she said, “The other children of his age are talking, what is wrong with your child?” Then I said there is nothing wrong with my child, nurses and doctors would have told me if there is a problem. But, eish, I do not understand because the child has been going to the well-baby clinic since birth. Then I took the child to the clinic and I was given a letter to go to a referral hospital. Later on, I was transferred to another hospital. But nobody was explaining to me what the problem with the child was.’ (P6, 28 years old, 1 child)

The study findings revealed that children were found to be having intellectual disability later in life and the diagnosis was not communicated well in advance to the mother.

#### Subtheme 3.1: Denial versus acceptance

Participants in this study displayed different feelings as far as the initial diagnosis of their children was concerned. Some mothers accepted the condition of their children, whereas others were in denial of the fact that their children were diagnosed with intellectual disability. Participant 7 (29 years old, 1 child) expressed her feelings saying, ‘It was a difficult task because I was not expecting this, and I said that it is not true, my child is not like that!’.

‘… The principal told me not to bring the child to school again as she must go to a special school. Then he told me what to do. I could not understand and believe what he was saying. The following day, I took my child to the same school again.’ (P5, 26 years old, 1 child)

Another mother added by saying:

‘I had this guilt feeling of saying “why me”, more especially that my husband left me also depresses me, but as I was going there time and again, bit by bit, I tried to accept. It was not easy at all. I had a lot of questions to ask myself, why this is happening to me, did I cause this, who might have caused this? I mean, as my first born child, I was expecting positive things and I was 26 years [*old*]. When I had my child, I planned to have him because I thought I am old enough to can have a child, but now, eish! [*crying*] He is always lying down and cannot do anything.’ (P12, 26 years old, 2 children)

Despite the challenges that mothers of children with intellectual disabilities were going through, they could cope and accept their children:

‘Yes, what I can say is that having a child with intellectual disability, you ought to accept this, because who do you think deserve to have an intellectual disability child? At first I thought I will never have another child again, but I just realise that I can still have a just not similar to this one [*without intellectual disability*]. At home there is no intellectual disabled child, but God gave me this child and I am proud of her.’ (P8, 26 years old, 2 children)

A mother verbalised her positive feeling and said:

‘I want my child to go to school. So that she can be able to do something for herself, because I will not live for her and live forever. I want her to be independent. I still believe that she can learn something or do handwork and make money out of it.’ (P11, 27 years old, 1 child)

## Discussion

This study intended to examine the challenges faced by mothers when parenting a child with an intellectual disability. Being a parent of a child with an intellectual disability cannot be compared to a parent of a child without a disability. Participants from this study displayed some frustrations, anger, guilt feelings, self-blame, isolation and discrimination. Their children were also called names and being treated like dogs. This behaviour or attitude adds more trauma and confusion to a mother who is already overwhelmed by the child’s condition. Anger, guilt feelings and frustrations have a negative impact on the care of a child, irrespective of whether a child has an intellectual disability or not. This is because these frustrations and anger are automatically transferred to the innocent child, who will in turn feel ignored and not taken care of. Therefore, the warm feelings and love from the mother are compromised, and consequently, the child’s growth will also be affected. Unfortunately, symptoms of guilt and self-blame can last for some years, depending on the different developmental milestones of a child and difficulties experienced (Stroebe, Schut & Boerner 2017). Mothers of these children expected more support from close family members like husbands and in-laws, which some of them never received. This poses further frustrations, as they were isolated rather than being supported. Mothers who were receiving support from their family members reported the support as not sufficient, as sometimes they were left alone by their husbands, especially when there are some disagreement (Marian et al. 2020). The birth of a child with an intellectual disability brings shock and shame to parents, especially the mother, and raising this child sometimes depends on one’s cultural belief (Riany, Cuskelly & Meredith 2016).

Mothers are always at the child’s side, although their husbands run away from them, avoiding the disappointment and embarrassment of the birth of such children in the families. This study concurs with that of McMahon et al. (2020), who highlighted that fear, anger and embarrassment are experienced by parents, especially of children who show signs of behavioural or physical disorders. A study done by Hoffmann, Windham and Anderson (2014) in California also revealed that mothers of children with autism spectrum disorders (ASD) have fewer subsequent children because of fear of having another child with ASD or any form of disability.

Mothers of children with intellectual disabilities in this study were experiencing financial problems. This was related to the care needed by a child. The financial needs include money to buy food, clothes and treatments, if not found at the health care facility. There is a lack of medication in the health facilities, especially public institutions. Patients are sometimes given prescriptions to go and buy medication from their own pockets, which they were rightfully supposed to be given for free. This practice tends to add more financial burden to the mother and family. Transport was also a problem when mothers accompany their children for check-ups. Different health care workers like occupational and physiotherapists were reviewing children on their own days of preference without looking at the appointments from the other health care workers. This practice further frustrates mothers, as they find themselves visiting the hospital twice or thrice a month, depending on the needs of the child. This becomes very expensive to them, as most of the mothers are not working. However, some practices tend to create unintentional frustration for parents of children with intellectual disabilities (Aston, Breau & MacLeod 2014). This study was supported by Osborn, Roberts and Kneebone (2020), who highlighted that mothers of children with intellectual disabilities experienced financial burden more than mothers of children without intellectual disabilities. The age of the child also poses a challenge, as the mother will be forced to put her child on her back and travel a long distance, approximately 3 km – 4 km or even more, to the health facility or to the taxi rank. In another study by Ouyang et al. (2014), financial burden was revealed to be because of the reduced income, as they need more time to care for their children. The latter study further indicates that mothers of children with intellectual disabilities work fewer paid hours, which results in a lower income than mothers of children without disabilities.

Some mothers explained that there was no proper communication, and they were not given a full explanation as to what was going on with their children when they were still at the hospital when their child was admitted. Health care professionals were said to be not giving enough information and support to the mothers after assessment. Tait et al.’s (2016) study is in line with the results of this study by revealing that parents who had the opportunity of knowing that there was a possibility of a child being diagnosed by professionals suspecting developmental disability were less likely to be shocked at the time of diagnosis. Mothers need to know everything about their children for them to understand and accept the child well. Furthermore, the study revealed that health care professionals lacked patience, support and enough time to explain to parents about the child’s diagnosis. They did not bother about the parents’ feelings or give them time to ask questions or talk about their feelings as a whole. This could be because of staffing shortages or a lack of counselling skills on the part of health care professionals at that time. However, Patel et al. (2020) in their research study indicated that the diagnosis of intellectual disability in children differs from age to age, and the type of intellectual disability can either be mild, moderate, severe or profound. On the other hand, the institutions lack counselling staff and there is a lack of referrals to appropriate health institutions in time. Some mothers were able to cope and accept their children, which was good as they would be able to assist those mothers who were still denying. Hence, a support group is essential for mothers of children with intellectual disabilities as it provides the most important information from the peers sharing or addressing their challenges as experienced (Collings et al. 2020).

### Limitation of the study

The study was conducted in the selected health institutions of the Vhembe district. It was limited to the hospitals and not the clinics or health centres. The study was limited to biological mothers, whereas experiences of caregivers and guardians who were staying and taking care of children with intellectual disabilities are not known.

### Recommendations

Based on the study findings, the researcher recommends that mothers of children with intellectual disabilities should be given full and accurate information on the child’s condition as early as it is discovered. This must include causes or predisposing factors, if any, treatment and prognosis. This will enhance the acceptance of the child and help them know what the child can and cannot do. Ongoing counselling is essential in the support of the mothers. Family counselling, including the siblings, is also important for the family to be able to support the mother, as she is not in isolation. Follow-up visits by different health care professionals must be done on the same day to avoid or reduce unnecessary expenses for the mother. Departments or offices like physiotherapy, social work and psychology must also be closer to outpatient department for easy access by the mothers to avoid moving around with a 12-year-old child on her back, for example. Mothers and their children with intellectual disabilities are to be loved, supported and accepted as they are, just like all human beings, as they are also human beings despite the fact that they have challenges facing them.

## Conclusion

The study revealed that support for mothers of children with intellectual disabilities is of vital importance and can be enhanced by proper communication. This might reduce unnecessary misunderstandings and confusion. Mothers were very upset to be told that their children had intellectual disabilities, as they were expecting a normal child without intellectual disability. Initial awareness provides a better understanding of the child’s condition by the mother.
